# Advances in photobiomodulation for cognitive improvement by near-infrared derived multiple strategies

**DOI:** 10.1186/s12967-023-03988-w

**Published:** 2023-02-22

**Authors:** Wei-tong Pan, Pan-miao Liu, Daqing Ma, Jian-jun Yang

**Affiliations:** 1 Department of Anesthesiology, Pain and Perioperative Medicine, The First Affiliated Hospital of Zhengzhou University, Zhengzhou, 450052 China; 2 Neuroscience Research Institute, Zhengzhou University Academy of Medical Sciences, Zhengzhou, 450052 China; 3 Henan Province International Joint Laboratory of Pain, Cognition and Emotion, Zhengzhou, 450000 People’s Republic of China; 4 Division of Anaesthetics, Pain Medicine & Intensive Care, Department of Surgery & Cancer, Faculty of Medicine, Imperial College London, Chelsea & Westminster Hospital, London, UK; 5 National Clinical Research Center for Child Health, Hangzhou, 310052 China

**Keywords:** Cognitive, Near-infrared light, Photobiomodulation, Nanoparticles, Photothermal

## Abstract

Cognitive function is an important ability of the brain, but cognitive dysfunction can easily develop once the brain is injured in various neuropathological conditions or diseases. Photobiomodulation therapy is a type of noninvasive physical therapy that is gradually emerging in the field of neuroscience. Transcranial photobiomodulation has been commonly used to regulate neural activity in the superficial cortex. To stimulate deeper brain activity, advanced photobiomodulation techniques in conjunction with photosensitive nanoparticles have been developed. This review addresses the mechanisms of photobiomodulation on neurons and neural networks and discusses the advantages, disadvantages and potential applications of photobiomodulation alone or in combination with photosensitive nanoparticles. Photobiomodulation and its associated strategies may provide new breakthrough treatments for cognitive improvement.

**Supplementary Information:**

The online version contains supplementary material available at 10.1186/s12967-023-03988-w.

## Introduction

Cognition is an advanced neurological function by which the brain acts in acquiring knowledge and understanding through thought, experience and senses. Cognitive dysfunction may occur due to various pathological or disease conditions and may impair learning and memory, accompanied by possible aphasia, apraxia, agnosia or dyslexia. Due to its refractory nature and harmfulness, the social impact of cognitive dysfunction is high [1]. Thus, research into the pathogenesis or risk factors for cognitive dysfunction in neurological diseases or conditions is urgently needed. More importantly, techniques and strategies that delay or prevent the onset and progression of cognitive impairment are urgently needed [2, 3].

Cognitive dysfunction is associated with regional abnormalities in different brain areas. Abnormalities such as neuroinflammation and impairments in neural network connectivity occur in many neurological diseases, including following surgery [4–9]. Although conventional treatments to improve regional blood supply were undertaken, such as medications or, in certain cases, craniotomies in patients with carotid artery stenosis, the effects or outcomes were unsatisfactory because of the unavoidable side effects of medication as well as the invasive trauma due to surgery. It has been suggested that developing nonpharmacological or noninvasive strategies to address cognitive disorders may offer better clinical outcomes for patients. Among these, conventional magnetic or electromagnetic fields, or light therapy to improve regional brain function, have been observed as alternative clinical treatments [10, 14].

Non-invasive techniques have unique advantages in the treatment of brain diseases due to the complexity of the cranial structure. Indeed, transcranial electrical stimulation (TES) and transcranial magnetic stimulation (TMS) have been used clinically and have achieved considerable curative effects [11, 12]. However, these techniques still have limited therapeutic efficacy because they lack targeted treatment for certain types of diseases and are not without complications, such as epilepsy [13]. To avoid adverse consequences, light therapy is now emerging as a new alternative treatment.

Photobiomodulation utilizes the photon energy of light to regulate the physiological functions of humans or animals [14]. Near-infrared (NIR) laser (780–1100 nm), which can effectively penetrate organs, including brain, has been studied for this application [15, 16]. Due to the complex structure of the brain and the diversity of disease conditions, NIR laser with different wavelengths, energy densities (expressed as J/cm^2^) and irradiance (expressed as mW/cm^2^) was selected for use. The most common are infrared bands around 800 nm and 1000 nm. However, in order to achieve better target on neural activity of the specific brain region and to obtain the maximum benefits, the frequency and irradiation time were varied at the experimental level. The energy generated from the absorption of photons by cellular mitochondria modulates the microenvironments of organisms to provide the capacity to treat disease or disease conditions. Moreover, the photothermal conversion effect triggered by photothermal nanomaterials endows photobiomodulation with accurate and effective features [17].

**Fig. 1:**
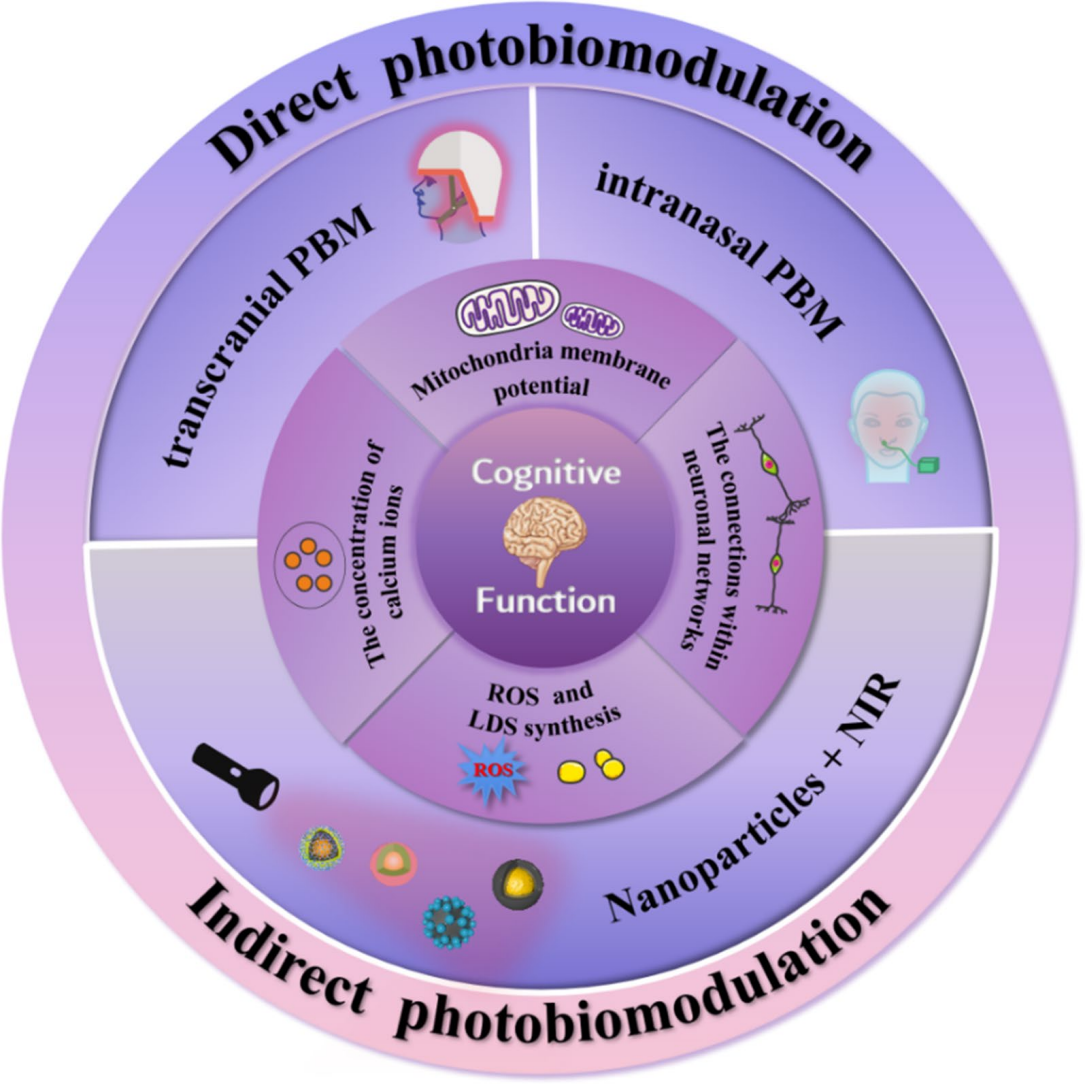
A summary of photobiomodulation approaches for the cognitive improvement. The photobiomodulation approaches for the cognitive improvement can be classified as the direct photobiomodulation, including transcranial or intranasal photobiomodulation, or and the indirect photobiomodulation, in conjunction with photosensitive nanomaterials. Both photobiomodulations modulate approaches are mainly based on the four pathological mechanisms to intervene in the physiological condition of cognitive function, including: mitochondrial function, calcium ion concentration, reactive oxygen species and neural networks. and Intervention consequently improves cognitive impairment related to various neurological diseases related cognitive impairment

This review focuses on NIR-based photobiomodulation (Fig. 1), including direct photobiomodulation and indirect photobiomodulation mediated by photosensitive nanoparticles, in improving cognitive function affected by various neurological diseases at the preclinical and clinical levels. This review discusses the underlying mechanisms of how photobiomodulation modulates neurons and neural networks and addresses the advantages, disadvantages, and potential applications of photobiomodulation alone or in combination with photosensitive nanomaterials.

## NIR Light Triggered Photobiomodulation

Photobiomodulation was formerly known as a low-level light therapy [18]. This therapy utilizes nonionizing light sources, such as lasers, light emitting diodes (LEDs) or broadband light, to generate ultraviolet, visible, and infrared light for therapeutic applications. The medical benefits of this low-level laser therapy were first proposed by Dr. Endre Mester in 1967. The studies by Zivin et al. verified that when a shaved scalp was irradiated with NIR laser (808 nm), the penetration depth of the cerebral cortex reached 20 mm [19]. Moreover, the light scattering effect of NIR laser is weak; it can penetrate deeper into the living tissue and does less harm to the organism [20–22]. Owing to these advantages, NIR-based photobiomodulation has been widely applied to alleviate pain or inflammation, modulate immune function, promote wound healing and promote tissue regeneration. By benefiting from these functions, photobiomodulation has been studied and applied in the field of neurotrauma, neurodegeneration and neuropsychiatric disorders [23–25].

Transcranial photobiomodulation is a general type of photobiomodulation in which light penetrates the skull into the brain matter to provide an effect [26]. During this process, light passes through a series of layers, including the scalp, periosteum, cranium and meninges, and induces neurobiological changes in turn [27, 28]. In the field of cognition-related disorders, studies have shown that transcranial photobiomodulation improves executive performance, memory, attention and other cognitive abilities, indicating that transcranial photobiomodulation is a potential therapy for the neurorehabilitation of cognitive function [29].

Intranasal photobiomodulation is another type of photobiomodulation that is an alternative to transcranial photobiomodulation because it overcomes some limitations of transcranial photobiomodulation and provides effective irradiation into certain brain regions, such as the ventral frontal lobe, ventral preorbital cortex and hippocampus [14, 30]. Intranasal photobiomodulation improves cerebral function due to its therapeutic mechanisms obtained from photothermal conversion, which modulates haemodynamic rheology, blood viscosity and coagulation function in regions where light radiation has been applied. Repeated intranasal photobiomodulation has been reported to potentially improve cognitive function [31, 32].

Integrating nanomaterials with photobiomodulation is another advanced type of photobiomodulation. The advantage of nanomaterial-integrated photobiomodulation is increased accuracy in treating brain region-related diseases. The combination of nanodrug-carrying particles and NIR laser is a forward-looking step in the development of photobiomodulation. Using the targeted drug-carrying ability and biocompatibility of nanomaterials, combined with the optical stimulation of an NIR laser, localized and timely release of nanodrugs in the brain can be achieved. This is of great benefit in visualizing the precise brain area/region that is being targeted and saves healthy brain tissue from suffering additional damage. Therefore, nanomaterial-integrated photobiomodulation has enormous potential for cognition-related diseases such as depression and Alzheimer's and Parkinson's diseases involving specific encephalic regions [33–35, 127]. Compared with transcranial photobiomodulation, intranasal photobiomodulation or even drug treatment, nanomaterial-integrated photobiomodulation may have more advantages for treating cognitive dysfunction; however, nanomaterials have not yet been deemed suitable for clinical use.

The effectiveness of photobiomodulation is significantly correlated with short-term cognitive improvement, but the long-term benefit of photobiomodulation is limited [36]. Indeed, a single dose of photobiomodulation was reported to improve short-term cognitive function [37]. In addition, the therapeutic effect of photobiomodulation was time-dependent: A study showed that the use of NIR laser (1064 nm, 250 mW/cm.^2^, 8 min) once a week for a total of 5 weeks can improve the behavioral cognitive processing of middle-aged and elderly subjects at a risk of cognitive decline. Although the duration of efficacy of photobiomodulation and its relapse are unclear, the extremely low adverse reactions and very promising outcomes in reducing the impact of cognitive decline were noted [38].

## Cerebral regulatory mechanisms of photobiomodulation

**Fig. 2:**
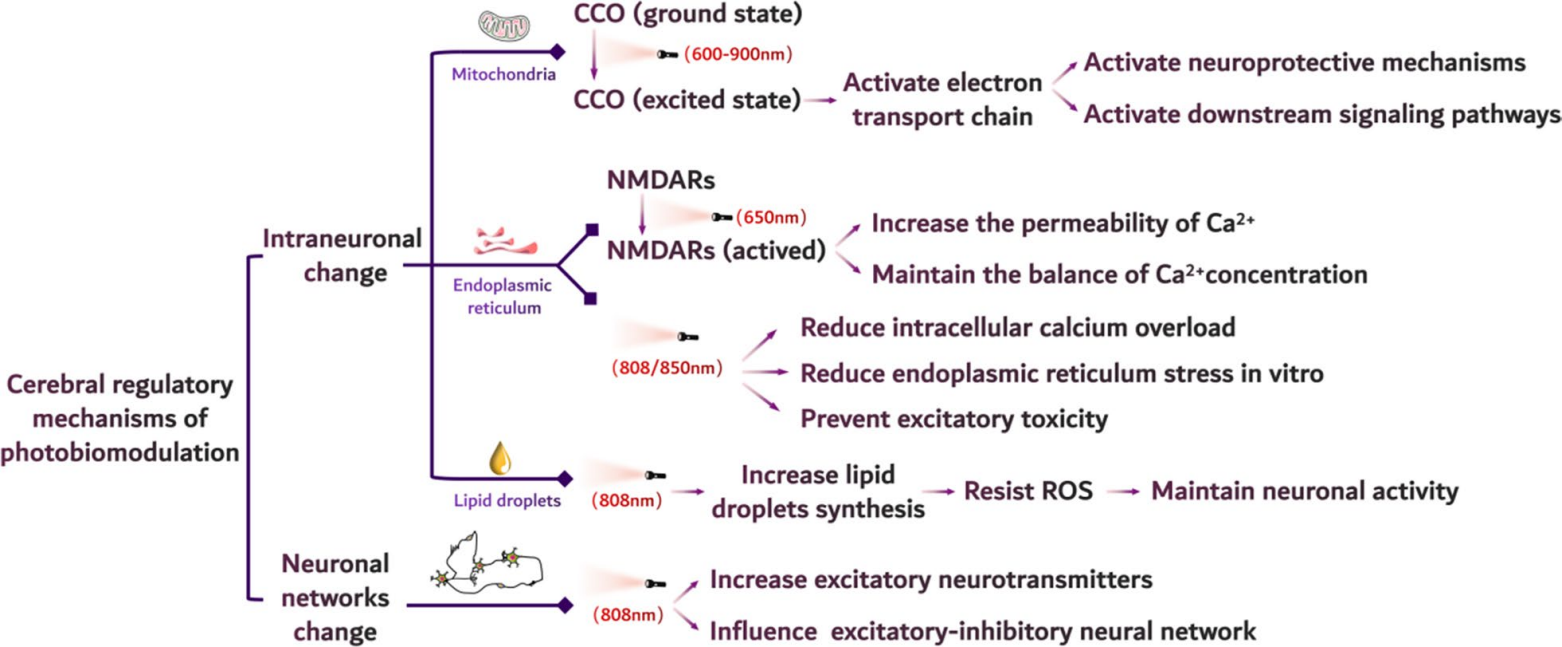
Biological mechanisms of near-infrared (NIR) light irradiating brain. They include cerebral functional states after intracellularly and intercellularly regulatory mechanisms have been applied and when neurons are irradiated by NIR light. Intracellular changes include cytochrome C oxidase, N-methyl-D-aspartic acid receptor and reactive oxygen species mediated mechanism whilst extracellular changes are enhanced neuronal transmission and connections

Photobiomodulation triggers neuroprotective mechanisms through a cascade of intracellular and molecular modulations, including increasing cerebral blood flow (CBF), balancing cellular metabolism and preventing neurodegeneration [39]. Red and NIR light were reported to alter intracellular molecules and neural network connections through the various mechanisms described above and below (Fig. 2) [40–43].

### Modulation of intracellular molecules

#### Cytochrome C oxidase

The main mechanism of light action on cells is to induce cytochrome C oxidase (CCO) located in the mitochondrial inner membrane, which is responsible for catalysing the transformation of oxygen into water for the production of adenosine triphosphate (ATP) [44, 45]. Biological tissues naturally contain chromophores that can be excited by light energy, and CCO is considered to be another major chromophore, alongside haemoglobin and myoglobin. CCO can be excited by light ranging in wavelength from 600 to 900 nm, which is why blue or green light was not selected for photobiomodulation despite these colours having the ability to promote cell growth [46]. Cells absorb optical energy, causing the redox state of CCO to change from a ground state to an excited state, which then activates the mitochondrial electron transport chain [40]. Elevated proton gradients on both sides of the mitochondrial membranes increase membrane potentials [47], and subsequently generate ATP and reactive oxygen species (ROS) required for normal cellular respiration, [48, 49] and enhance cell energy consumption [50]. Subsequently, downstream signalling pathways were activated to protect neurons, to promote neuronal proliferation and to form new synapses for better memory formation [49, 51].

#### Intracellular calcium ions (Ca.^2+^)

Visible-NIR light has also been reported to increase the permeability of neurons to calcium ions (Ca^2+^) and maintain neuronal Ca^2+^ intra- and extracellular balance [52, 53]. Both the entry of exogenous Ca^2+^ into cells and the release of endogenous Ca^2+^ from the endoplasmic reticulum of neurons is due to the activation of N-methyl-D-aspartate receptors (NMDRs) once membranes are depolarized by irradiation with 650 nm and 808 nm light. Ca^2+^ is involved in the regulation of neurotransmitter release, synaptic plasticity, and activity-dependent transcription under normal physiological conditions, but over-influx of calcium may also cause cytotoxicity [41]. Low-intensity NIR irradiation (e.g., 850 nm laser radiation) has been reported to increase intracellular Ca^2+^ levels and protect against the effects of intracellular calcium overload and endoplasmic reticulum stress in vitro [54]; all these ultimately protect cells from the destructive effect of high Ca^2+^ levels, promote the excretion of intracellular calcium ions, and prevent excitatory toxicity (810 nm laser radiation) [55].

#### ROS

NIR irradiation generates ROS, and then modulates the metabolic activity of neurons by altering lipid metabolism [49]. During the neuronal metabolism, the accompanying synthetic lipid droplets (LDS) mitigated the harmful effects of ROS through enveloping and coating mechanisms [56, 57]. Additionally, photobiomodulation can balance the intracellular ROS levels in a steady state through reducing the increased intracellular ROS levels in stressed neurons but increasing ROS levels in normal unstressed neurons due to low level laser triggered mitochondrial membrane potential changes [58]. A previous study also showed that after 808 nm laser irradiation, the average lipid as well as LDS levels of rat cortical neurons were increased significantly mediated by ROS, indicating a strong correlation between ROS induced by photobiomodulation and LDS formation in neurons. This also means that the lipid metabolism of neuronal cells can be manipulated through NIR laser to treat cognitive disturbances [49].

### Neuronal networks

Transcranial photobiomodulation has an inhibitory effect on rodent brains because light irradiation reduces the increased excitatory neurotransmitters in the hippocampus and cortex [59]. This suggests that transcranial photobiomodulation is an effective intervention strategy for excitatory/inhibitory neurons as well as the entire excitatory/inhibitory neural network. Recently, a preliminary study on the regulatory effects of 40 Hz pulsed NIR laser on brain oscillations demonstrated that a single session of transcranial photobiomodulation significantly increases the power of electroencephalogram (EEG) faster oscillatory frequencies of alpha, beta and gamma waves and reduces the power of the slower frequencies of delta and theta waves in subjects at rest [43]. It is suggested that infrared light can maintain the stability of the brain neural network [26, 60]. Transcranial photobiomodulation with an 808 nm pulsed NIR laser (transcranial: 100 mW/cm^2^; intranasal: 25 mW/cm^2^: 40 Hz for 20 min) was recently reported to reduce neuronal damage in the prefrontal cortex and γ-aminobutyrinergic (GABAergic) neurons in the hippocampus, protect the integrity of the perihippocampal inhibitory network composed of parvalbumin-positive neurons, and maintain the normal hippocampal γ band rhythm [43]. As an energy wave, light can affect the functional connectivity of neural networks by affecting the function and state of certain neurons, such as inhibitory neurons, resulting in the remodelling of oscillatory frequencies in large-scale neural networks [39].

## Photobiomodulation promoting cognition

The potential applications of photobiomodulation have been explored in many disorders, ranging from neurotrauma and neurodegeneration to neuropsychiatric disorders. Photobiomodulation can modulate human/animal brain function and is a new potential treatment strategy for neurological diseases.

### Transcranial photobiomodulation

Transcranial photobiomodulation has been studied in different animal models and humans as an economical and safe therapy for cognitive dysfunction [44, 61].

#### Modulation in healthy human brain

Several studies have reported that transcranial photobiomodulation enhances cognitive function by regulating the electrical activity of the healthy human brain (Table 1). The manifestations of cognitive function, including sleep quality and emotional state, can be explored using EEG patterns. In particular, memory and attention were significantly improved after application of photobiomodulation to the human brain. In addition to the molecular mechanisms mentioned above, the effect of transcranial photobiomodulation on brain tissue is also related to improvements in CBF [62]. Transcranial photobiomodulation applied to the prefrontal cortex not only can activate CCO but can also improve cerebral oxygenation and increase blood oxygen content and CBF, all of which are necessary for the high-level energy demands needed to maintain normal cognitive function [63].

**Table 1:** Studies on improvement of cognitive function in healthy people after transcranial photobiomodulation

Study (year)	Ages	Wavelengths and Irradiation Parameters	Irradiation Approach and Sites	Outcomes or Findings
Chang et al. (2012) [68]	20–21 years old	830 nm; 7 mW per diode, 20 J/cm^2^, 10 min, PW at 10 Hz with DC of 50%	Remote tissue irradiation; 1 site, left palm	Laser stimulation at 10 Hz can increase the power of alpha rhythms and theta activities, EEG can be interfered
Barrett and Gonzalez-Lima (2013) [69]	18–35 years old	1064 nm; 250 mW/cm^2^, 60 J/cm^2^, 4 min, one irradiation session, CW	Transcranially; 2 sites, unilateral (right frontal pole on 4 cm medial and lateral)	After 2 weeks of irradiation, subjects have positive emotional states, significant improvement of both attention and memory
Gonzalez-Lima et al. (2015) [70]	Mean age: 20.4 years	1064 nm; 250 mW/cm^2^, 60 J/cm^2^, 8 min, one irradiation session, CW	Transcranially; 2 sites, (lower and upper portion of right lateral forehead at EEG map sites: FP2, F4	Improved executive function
Gonzalez-Lima et al. (2016) [71]	17–35 years old	1064 nm; 250 mW/cm^2^, 60 J/cm^2^, 8 min, one irradiation session, CW	Transcranially; 2 sites, lower and upper portion of right lateral forehead at EEG map sites: FP2, F4, and F8 site	Improved prefrontal rule-based learning, and increased learning related cognitive function
Salimi et al. (2018) [29]	Males:19–25 years Females:18–25 years	850 nm; 285 mW/cm^2^,60 J/cm^2^, 2.5 min, 1.4 cm^2^, CW	Transcranially; On the right prefrontal cortex, especially Fp2 region	After photobiomodulation, attention and alertness are improved, cognitive function is enhanced, and delta power is decreased simultaneously
Chan et al. (2019) [37]	Mean age: 66.2 years	870 nm; 44.4 mW/cm^2^, 7.5 min, 22.48 cm^2^ and 999 mW totally	Transcranially; 3 sites; Left frontopolar at FP1 and right frontopolar at FP2 and Pz	The older adults who received photobiomodulation exhibited significant improvements in the frontal brain functions, such as action selection, inhibition ability, and mental flexibility

#### Modulation in cognitive dysfunction

Compared with the photoregulation of normal human cognitive function, the use of transcranial photobiomodulation to treat brain diseases is still limited. However, in recent years, transcranial photobiomodulation has shown potential for promising applications in the treatment of cognitive impairment in traumatic brain injury (TBI) and Alzheimer's disease (AD).

##### Alzheimer's disease

The onset of AD in patients is often accompanied by symptoms of cognitive impairment. Early studies found that red lasers emitted from an arterial duct can irradiate the brain, improve CBF in AD patients and lead to significantly lower dementia scores [64]. Later, researchers found that photobiomodulation can also affect cellular signalling pathways or neuronal network oscillations, which are more closely related to the pathophysiology of cognitive dysfunction [43]. Recently, amyloid-beta protein (Aβ), the most important pathological brain indicator in the brains of AD patients, has become a target for transcranial photobiomodulation therapy. Photobiomodulation was found to accelerate Aβ degradation while reducing Aβ accumulation and microglial proliferation, subsequently improving cognitive impairment [34, 65, 66]. Recently, transcranial photobiomodulation was applied to AD patients, and their cognition was improved [67]. Studies and trials on transcranial photobiomodulation in the treatment of AD are summarized in Table 2. These studies may lay a foundation for the use of transcranial photobiomodulation in treating AD in the future.

**Table 2:** Studies on improvement of cognitive dysfunction in AD patients after transcranial photobiomodulation

Study (year)	Mode	Wavelengths and Irradiation Parameters	Irradiation Approach and Sites	Outcomes or Findings
Maksimovich et al. (2015) [64]	AD patients (34.83% male and 65.17% female) without serious comorbidities	Visible region of spectrum; 20 mw, fiber diameter of 25–100 μm, 20–40 min	Transcatheterly	Improved cerebral microcirculation and cognitive recovery; decreased permanent dementia
Nichols et al. (2017) [62]	AD patients	1064 nm, 3.4 W, 250 mW/cm^2^, 120 or 137.5 J/cm^2^ per session, CW	Transcranially	Improved reaction time and lapses in psychomotor vigilance task and correct responses in delayed match to sample task; increased resting-state EEG alpha, beta, and gamma power;
Glushkovskaya et al (2019) [24]	Mongrel male mice injected Aβ (1–42) peptide (1 μL, 200 μmol) in the CA1 field of the hippocampus bilateral	1267 nm, 50–200 mW/cm^2^, 18–39 J/cm^2^ at brain’s surface, beam diameter of 5 mm, CW	Transcranially; High power laser diode (LD-1267-FBG-350)	Transcranial photobiomodulation significantly reduces the deposition of Aβ plaques (by stimulating lymphatic drainage) and improves cognition, memory and neurological status
Xing et al. (2020) [72]	Double transgenic mice (APPswe/PSENdE9)	632.8 nm, 92 mW, 10 min, spot area of 0.785 cm^2^ in cerebral cortex and hippocampus, once a day, CW	He–Ne laser	Transcranial photobiomodulation has the ability to improve cognitive impairment in Alzheimer’s disease
Shin et al. (2020) [73]	Transgenic (5XFAD) male mice	610 nm, 1.7 mW/cm^2^, 2.0 J/cm^2^, spot diameter of 4 mm onto the midpoint of the parietal bone and the posterior midline of the seventh cervical vertebra, 20 min × 3, 14 weeks	Skin-adherent LED probe	Transcranial photobiomodulation has the ability to reduce amyloid accumulation, neuronal loss and microgliosis and improve cognitive impairment by elevating insulin-degrading enzyme associated with Aβ degradation
Huang et al. (2021) [67]	AD patients with dementia but without serious comorbidities	1060–1080 nm and 15,000 mW, 23.1 mW/cm^2^, ~ 650cm^2^ per treatment area, 6 min × 2 daily for 8 weeks	Transcranially (light treatment helmet devices)	The NIR light treatments demonstrated safety and positive cognitive improvements in AD patients with dementia, and the trial designed a simple treatment that could benefit dementia patients

##### Traumatic brain injury

Compared with AD, there are more cases that have used transcranial photobiomodulation treatments in cases of TBI in the past, but the studies regarding cognitive impairment were mainly animal studies (Table 3), likely due to the following reasons: (1) The animal models are, in essence, acute TBI models, while clinical TBI patients are usually chronic, and the mechanisms behind cognitive impairment are different [77]. (2) Furthermore, light decays exponentially when it passes through the skull and brain tissue. Studies have shown that NIR laser may not penetrate the human brain deeper than 20 mm from the cortical surface, [78] while the location of TBI brain damage is usually deeper; hence, the effect of transcranial photobiomodulation in treating TBI is not as effective as that of AD.

**Table 3:** Studies on improvement of cognitive dysfunction in TBI models after transcranial photobiomodulation

Study(year)	Mode	Wavelengths and Irradiation Parameters	Irradiation Approach and Sites	Outcomes or Findings
Whalen et al (2012) [66]	Male C57BL/6 mice, 3 months of age weighing 25–30 g	800 nm; 250–1000 mW/cm^2^,60–210 J/cm^2^, spot area of 1.32 cm^2^, 2 or 7 min, CW	A) Via an open craniotomy; Holding probe at 1 cm above head B)B)B)B)B)Transcranially; At the right and left parieto-temporal region	Improved cognitive performance in MWM test (at 60 J/cm^2^ by transcranially or via an open craniotomy); decreased microglial activation at 48 h post-TBI
Henderson et al. (2015) [74]	Lamb heads, TBI patients	810 and 980 nm; 10 and 15 W, 14.8–28.3 J/cm^2^, 8–12 min per site, 2–3 times/week for 8 weeks, PW at 10 Hz	Transcranially; bilateral forehead, bilateral prefrontal and temporal	Improved symptoms of headache, sleep disturbance, cognition, mood dysregulation, anxiety, and irritability
Hamblin et al. (2016) [75]	Male BALB/c mice, aged 6–8 week weighing 20–25 g	810 nm; 25 mW/cm^2^, 18 J/cm^2^, spot diameter of 1 cm, 12 min, for 3 or 14 days, CW	Transcranially; Covered the entire skull	By 3 times a day: improved neurological severity score; improved cognitive performance in MWM test; decreased lesion size (at 2–8 weeks post-TBI); increased expression of GFAP in perilesional cortex, Dentate Gyrus and SVZ (at 8 weeks)
Micci et al (2018) [23]	Male Sprague–Dawley rats (200–350 g)	808 nm; 10 Nano second pulsed laser (NPLT), 20 Hz, 15 mJ/pulse, 5 min, 300 J/cm^2^ totally	Transcranially; A 3 mm diameter specially developed fiber-optic bundle system	Transcranial photobiomodulation significantly reduces neuronal death and inflammation, increases neurotrophin expression and proliferation of neural progenitors in the hippocampus, and improves vestibulomotor and cognitive functions
Micci et al (2020) [76]	Male Sprague–Dawley rats (350 g–400 g)	808 nm; 10 Nano pulsed laser (NPLT), 15 mJ/pulse, 5 min, 20 Hz, 300 J/cm^2^ totally	Transcranially; A 3 mm diameter optical fiber	TBI models' cognition and neurogenesis can be mitigated. NPLT significantly decreased aberrant migration of neural progenitors, while preventing TBI-induced upregulation of specific microRNAs (miRNAs) in NSC

At the molecular level, transcranial photobiomodulation conducted in the treatment of cognitive impairment in TBI animal models decreased inflammation and neuronal death, increased neurotrophic factor expression in the hippocampus and neural progenitor cell proliferation, and overexpressed protruding proteins. In terms of brain function, the effects included reductions in lesion size, improvements in cognitive function and reductions in anxious behaviour. Clinically, transcranial photobiomodulation was applied to patients with TBI to improve symptoms, such as relief of headache symptoms, enhancement of sleep quality, and improvement of cognitive and emotional state [73].

#### Other applications

Transcranial photobiomodulation has been considered to treat other cognitive impairment-related brain diseases [79]. A few studies reported that transcranial photobiomodulation was used to treat Parkinson's disease (PD), the positive effects of which were related to irradiating tyrosine hydroxylase positive (TH +) neurons in the substantia nigra pars compacta (SNc) to improve disabling dyskinesias [80–83]. In a recent prospective proof-of-concept study, the cognitive performance of PD patients was improved after both 12 weeks and 1 year of treatment with a device that combined transcranial photobiomodulation and abdominal photobiomodulation [81]. Moreover, a study of transcranial photobiomodulation for autism spectrum disorder (ASD) in children and adolescents aged 5–17 years found that low-level laser therapy reduced irritability and other symptoms and the behaviours associated with ASD, with these positive changes maintained and augmented over time [84]. Because of its high safety, few side effects and low cost, transcranial photobiomodulation is a potential therapy for other disease conditions, such as depression or pain-related cognitive dysfunction, but warrants further study.

### Photosensitive nanoparticles in photobiomodulation

Because of its high safety, few side effects and low cost, transcranial photobiomodulation is a potential therapy for other disease conditions, such as depression or pain-related cognitive dysfunction, but warrants further study [85].

The common method in combination is to use 10–100 nm nanoparticles constructed by biocompatible materials as carriers to carry the drug to increase the amount of drug targeted at tissue or cells and greatly improve the pharmacological bioavailability locally [86]. Nanodrug delivery systems need to have the following basic characteristics: in addition to being nontoxic, harmless and degradable, drugs must be able to be selectively transported across the blood brain barrier (BBB) after administration and have the ability to evade the immune system in vivo. Drug transport must be targeted to release sufficient amounts of drugs to specific brain regions [87].

Currently, more advanced nanodrug delivery systems, known as “intelligent” nanoparticles, are emerging on the basis of incorporating materials science. The modified nanoparticles respond to some stimuli due to enhancements of special materials to achieve spatially or temporally controllable release. The types of stimuli can be physical, chemical or biological [88]. Chemical and biological stimuli are generally internal, for example, the effects of different biomolecules, pH values, and redox reactions in organelles within cells, while physical stimuli are generally external, including external magnetic fields, electric fields, temperature and illumination [89]. In contrast to traditional administration, nanomaterials improved drug release and minimized the drug dose required to be effective. The outstanding advantage of modified nanoparticles is that the spatiotemporal mechanism of drug release is manually controlled, which is more practical and convenient for more effectively achieving the desired effect [90].

Among various “intelligent” nanodrugs, photosensitive nanoreagents have attracted the most attention due to their photothermal conversion effects and unique ways of realizing on-demand drug delivery [91]. Compared with other wavelengths of light, NIR laser is not easily absorbed by interfering chromophores and penetrates more deeply without causing tissue damage. Therefore, NIR laser has unique advantages in terms of noninvasive tissue penetration to achieve drug delivery, and thus, the combination of NIR laser with photosensitive nanoparticles may provide hope for the future [92, 93]. NIR laser with wavelengths of 700–1000 nm is usually selected to combine with different types of nanocarriers for drug delivery [94]. Because using a single type of nanometre material makes it difficult to meet the multiple needs of drug delivery and NIR light response, researchers are currently working on nanoparticles made of composite nanomaterials, which can not only maintain the desired levels of drugs in the nanoparticles but can also ensure that the drugs are not destroyed by an immune response. The most important feature is that they have good temporal and spatial controllability because of NIR irradiation. Based on these requirements, a variety of composite nanoparticles have been developed, among which photosensitive materials show physical or chemical transformation upon exposure to NIR radiation. Then, the original structures of the nanoparticles are destroyed, and the loaded drug is released. The emergence of these photosensitive nanoparticles may provide new approaches for treating neurological diseases.

**Fig. 3:**
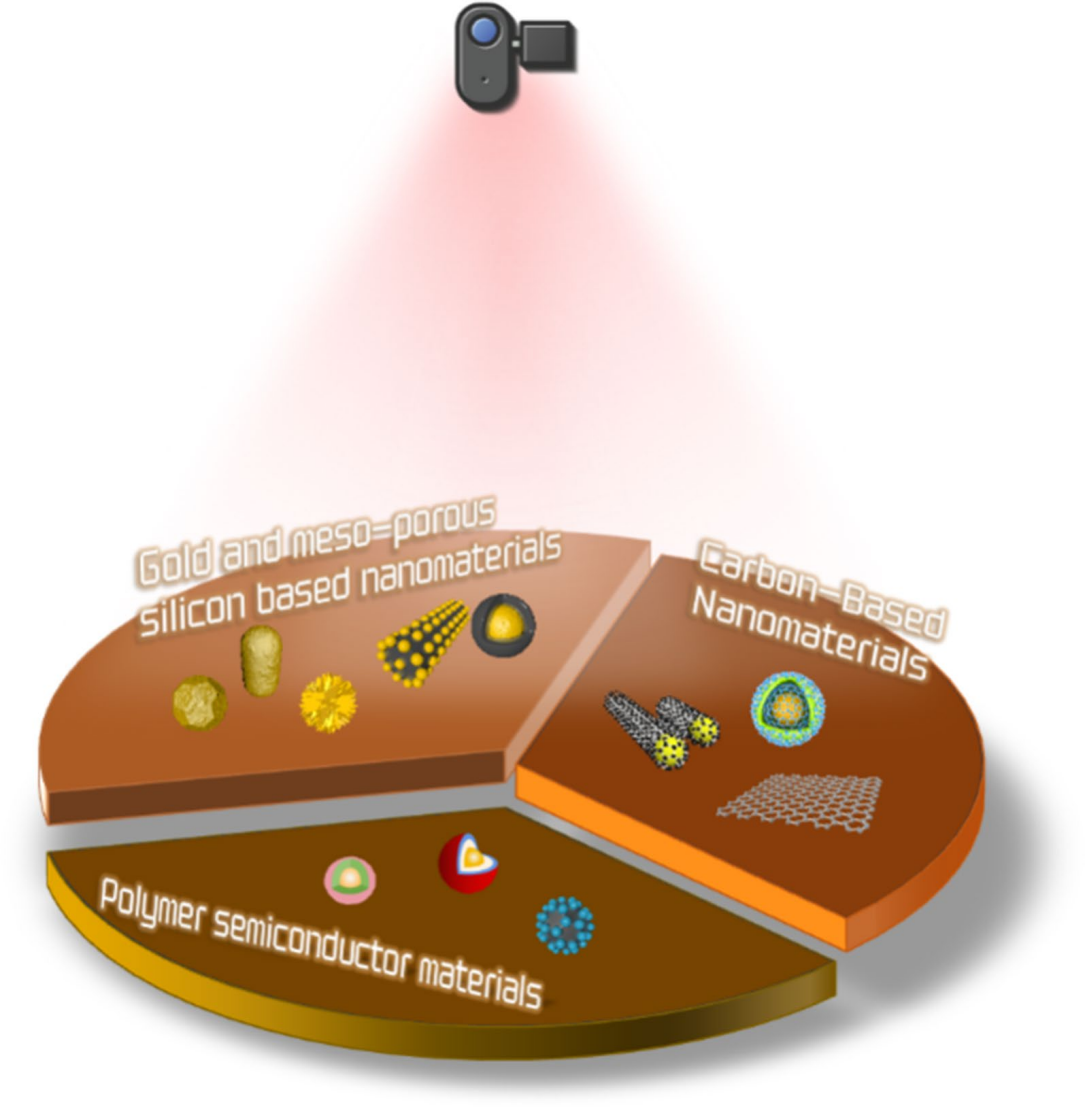
Photosensitive nanoparticles in combination with NIR laser for brain diseases. The photosensitive nanoparticles include gold-based nanoparticles (from left to right are gold nanoparticles, gold nanorod and gold nanostar, gold-based liposomes and mesoporous silica nanoparticles with gold cores), carbon-based nanomaterials (from left to right are carbon nanotubes, graphene oxide and graphene quantum dots) and polymer semiconductor materials (from left to right are common semiconductor polymer nanoparticle, semiconductor polymer nanoparticle coated by red blood cell membrane, semiconductor polymer nanoparticle coated by photothermal materials such as phenyl di-n-pentylphosphinate)

Common NIR response nanomaterials that can be used in brain diseases consist of gold, carbon, and semiconductor polymers [95–97]. These materials can be used to construct nanoparticles by themselves or participate in the construction of nanocarriers as components. The resulting composite nanoparticles have the ability to transport drugs and produce photothermal conversion under NIR irradiation, thus improving related nanodrug delivery systems (Fig. 3). The following provides an overview of the applications of NIR-photosensitive nanoparticles in brain diseases in terms of the material types triggered by NIR laser [98–103].

#### Gold-based nanomaterials

In recent years, gold (Au) materials have become prominent in the field of nanomaterial research because of their high biocompatibility, easy synthesis and possibility of surface modification [95]. The advantage of Au is its ability to generate localized surface plasmon resonance (LSPR) under NIR irradiation, which is manifested as the collective oscillation/excitation of surface electrons, thus utilizing the absorption of light energy and photothermal conversion [104]. The good photothermal properties and BBB penetrability mean that Au is an excellent carrier for NIR-responsive drug delivery and for use in brain disease treatments [88, 105].

##### Au nanomaterials

**Fig. 4:**
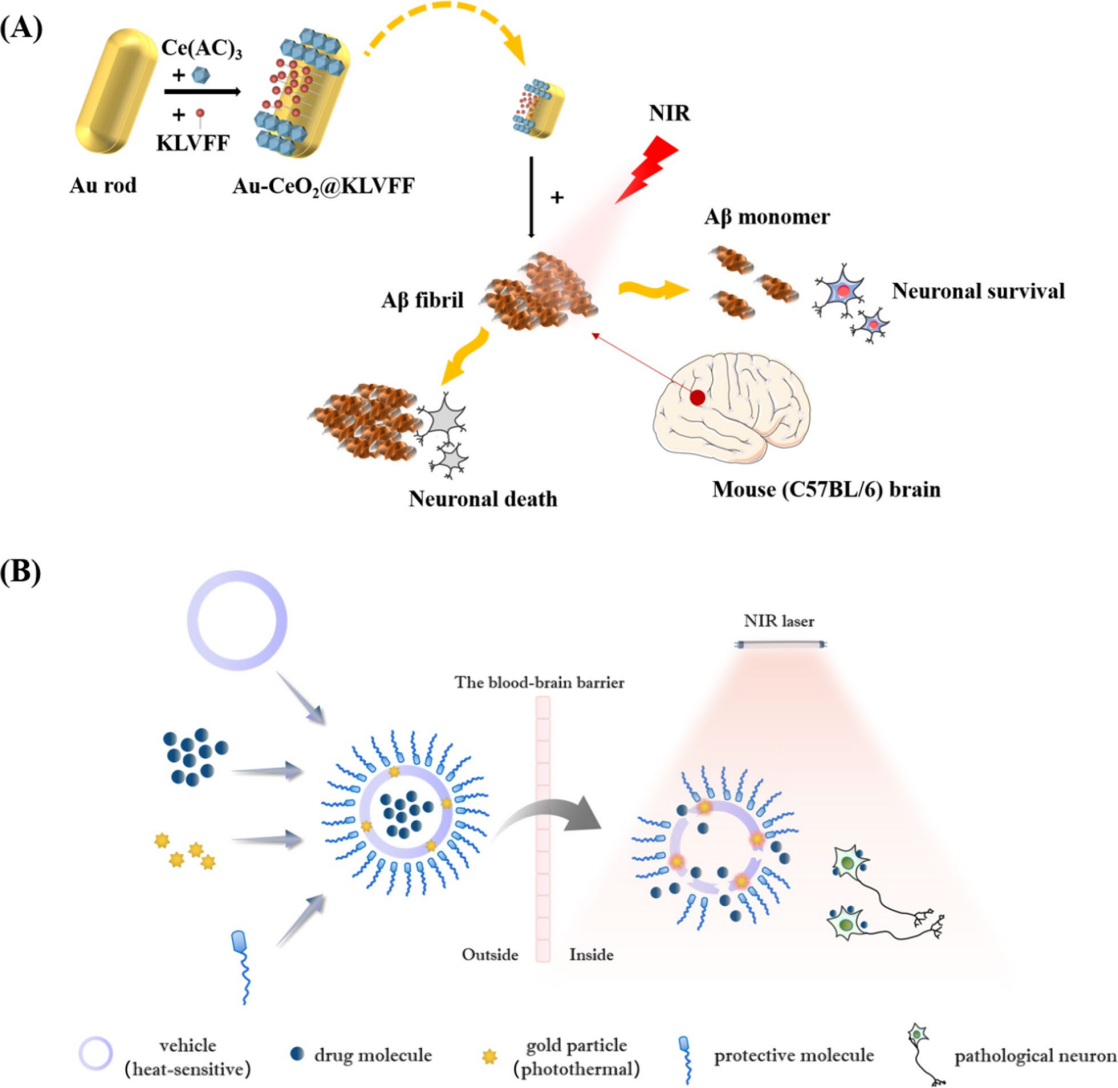
Scheme of photothermal gold nanoparticles and related application example. **A** Scheme of gold nanorod treating Alzheimer's disease. AuNRs can bind with CeO_2_ and Aβ-targeted inhibitory peptides to target Aβ fibrils through photocatalysis and photothermal effect, which was used in multi-modal therapy of Alzheimer’s disease [107]. Copyright 2022, American Chemical Society and its reproduced permission granted. **B** Structural pattern diagram of nanoparticles containing a photothermal material (gold). Composite nanoparticles modified with protective molecules can flow through the bloodstream and cross the blood–brain barrier. Under NIR laser irradiation, gold particles undergo photothermal conversion, and the heat can be generated to dissolve the heat-sensitive materials used to construct the vehicle, so that the drugs can be released to act on neurons

Gold nanomaterials with different structures can be obtained by adjusting the preparation process, including gold spheres, gold nanorods (AuNRs), gold nanoparticles (AuNPs) and gold nanostars (AuNSs). Among these, AuNRs, AuNPs and AuNSs are common pure gold-based nanoscale structures, and they have different advantages in interventions involving brain neurons. The direction of electron oscillation in AuNRs is a combination of transverse and longitudinal oscillations, which has higher NIR laser absorption than AuNPs and AuNSs. Shan et al. reported the NIR laser absorption properties of AuNRs loaded with single chain variable fragment (scFv) 12B4 and thermophilic acylpeptide hydrolase (APH) ST0779 as a smart theranostic complex (GNRs-APH-scFv, GAS), which possesses both rapid detection of Aβ aggregates and NIR light photothermal treatment that effectively disassembles Aβ aggregates and inhibits Aβ-mediated toxicity [106]. Moreover, researchers also coated both ends of AuNRs with CeO_2_ nanoparticles and introduced photocatalysis and photothermal therapy in the NIR laser into the AD treatment (Fig. 4A). The photothermal effect significantly improved the permeability of the BBB and overcame the shortcomings of traditional anti-AD drugs. To further improve the therapeutic efficiency, Aβ-targeted inhibitory peptides (KLVFF) were modified on the middle surface of AuNRs (K-CAC). The behavioural data showed that K-CAC improved the cognitive function of AD mice by degrading Aβ protein deposition and scavenging ROS [107].

**Fig. 5:**
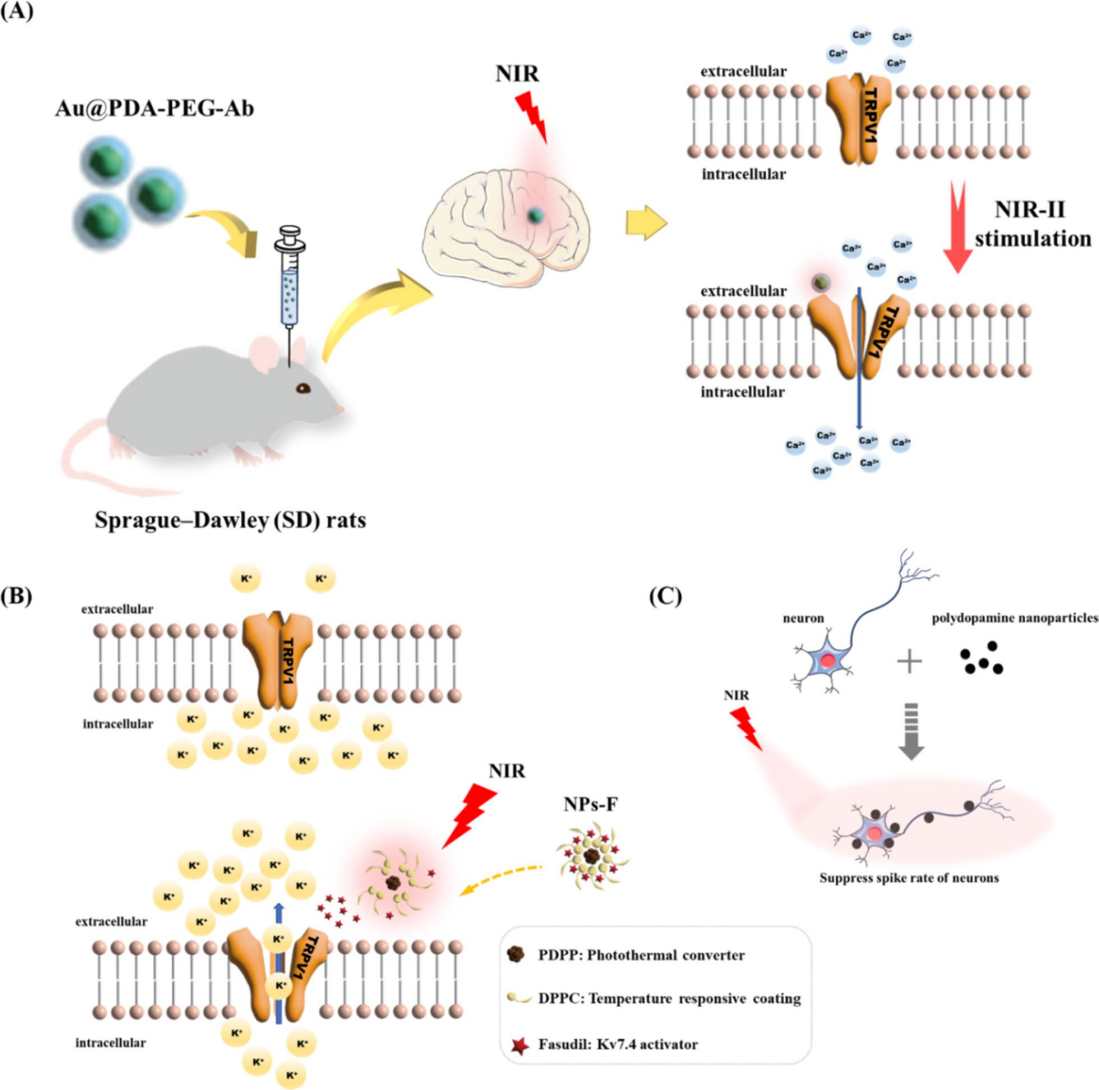
The examples of polymer-associated nanoparticles that affect neurons and ion channels’ activities. **A** Scheme of preparation of antibody-conjugated gold nanoparticles (Au@PDA-PEG-Ab) and mechanisms of nanoparticle-mediated NIR-II laser neural stimulation [109]. Copyright 2022, Springer Nature and its reproduced permission granted. **B** Schematic illustration of PDA-nanoparticles-mediated photothermal stimulation of neurons. PDA nanoparticles (PDA NPs) localized on the neuron modulate the neural activity through photothermal conversion of NIR light [128]. Copyright 2021, Wiley–VCH GmbH and its reproduced permission granted. **C** Schematic illustration of the preparation of nanoparticle (NPS-F) and the activation of Kv7.4 channel under NIR irradiation. The mechanism of reducing the excitability of ventral tegmental area dopamine neurons is also reported [126]. Copyright 2021, John Wiley and Sons and its reproduced permission granted

However, AuNPs and AuNSs have advantages that AuNRs do not have, such as smaller sizes, higher precisions of neuronal regulation and shorter synthesis times. Additionally, AuNSs, which have excellent biocompatibility, can also be directly attached to the membranes of neurons to regulate their activity [108]. In this context, Li et al. recently reported that gold-based photosensitive nanomaterials can be produced by coating gold nanoparticles with polydopamine and anti-TRPV1 antibodies (Au@PDA-PEG-Ab) **(Fig. ****5****A)**. To verify the photothermal effect on neurons, Au@PDA-PEG-Ab particles were cocultured with TRPV1 receptor-enriched HT-22 cells and then irradiated by NIR-II laser in vitro. The results showed that TRPV1 receptor-enriched HT-22 cells had a significant influx of Ca^2+^, while this was not seen in SH-SY5Y cells that lacked TRPV1 receptors. In vivo experiments further demonstrated that Au@PDA-PEG-Ab specifically excited pyramidal neurons of the hippocampus (5 mm deep in the brain parenchyma) when applied to rats [109]. These data indicate that gold-based photothermal nanomaterials may have specific regulatory effects on neurons enriched with TRPV1 receptors in the brain.

##### Au composite nanomaterials

In addition to the abovementioned nano preparations prepared with pure gold materials, Au can also be used as a photothermal material in the preparation of composite nanoparticles, and then loaded drugs or designed drugs can be released at scheduled times and positions under NIR radiation control. A common structural pattern of composite nanoparticles contains gold (Fig. 4B). Liposomes are the most successful nanocarriers used clinically [110], but the bioavailability of internally loaded drugs in liposomes is not ideal. The photothermal conversion effect of gold can change the microenvironment, destroy the stability of liposomes, and release internal drugs on demand. Therefore, the concept of developing nanoparticles combined with gold and liposomes (AuNP-liposomes) has attracted much attention [88, 111, 112]. To maintain the stability of liposome morphology, the sizes of metal particles should not be more than 6.5 nm [113]. AuNPs can be bound to liposomes by different methods, such as reverse evaporation (REV), thin film hydration (TFH), interdigitation-fusion, and lipid vesicle metallization. AuNP-liposomes induce enhanced permeability of the liposome membrane based on NIR irradiation and Au photothermal conversion ability [114]. Under irradiation, the heat caused by electron exchange in gold was absorbed by liposomes, resulting in membrane collapse or induced phase transformation of the lipid bilayer, and the loaded drug flowed out completely during the drug delivery process [115]. As early as 2008, researchers discovered the controlled release effect of an NIR pulsed laser on liposomes coated with gold nanohollow shells [116]. In 2016, the release of the model drug (PTX) from liposomes coated with AuNPs and AuNSs was performed by laser excitation at NIR wavelengths [117]. With regard to brain-targeted drug delivery, a recent study published in 2020 reported a type of transferrin (Tf) receptor-targeted, gold-based therapeutic liposome containing docetaxel (DCX) and glutathione-reduced gold nanoparticles (AuGSH) for brain-targeted drug delivery and imaging. The liposome provided a higher drug loading rate and co-encapsulated DCX for simultaneous targeted therapy [118].

These findings indicate that drugs, such as water-soluble medicines that are unable to permeate the BBB, can be loaded on different carriers, which can be composed of a single substance or compound. A composite nanocarrier has multiple properties based on different substances, thus contributing to nanodrug delivery platforms with different targets. If combined with NIR laser, these nanoparticles not only enhance the drug targeting ability but also improve the controlled release and bioavailability of the loaded drugs. Composite nanocarriers are of great significance for treating brain diseases because most drugs cannot pass the BBB, such as memantine, which improves cognitive function. Hence, the combination of NIR laser with nanocarriers helps drugs to pass the BBB while ensuring the release of drugs in assigned brain regions to maximize drug utilization and minimize drug toxicity.

#### Polymer semiconductor materials

Compared with biotoxic metal Au nanomaterials, semiconductor polymer nanoparticles (SPNs) have the advantages of a π-π electron delocalization framework, high extinction coefficient and excellent photothermal conversion efficiency and are more suitable as phototherapy agents for treating brain diseases [119, 120]. SPNs are nanoparticles that have been developed for photobiomodulation over the years [121, 122]. The advantages of semiconductor polymer materials are as follows: (1) completely organic, inert and nontoxic in vivo, showing ideal biocompatibility for organisms [123]; (2) flexible synthesis and convenient preparation [124], and (3) good photostability and excellent photothermal properties [125].

SPN combined with NIR laser has been found to regulate neuronal activity (Fig. 5B). Singamaneni et al. demonstrated that polydopamine nanoparticles are biocompatible and biodegradable photothermal sensors that can suppress the spike rate of neurons in animals under NIR laser irradiation with different power densities. The data showed that when the power density was 3 mW/mm^2^, the spike rate of neurons decreased by 39%. When the laser power density was increased to 6 mW/mm^2^, the spike rate decreased to 98%, suggesting that neuronal activity was almost completely shut down under such irradiation conditions. Similarly, NIR irradiation at different times also inhibited the spike rates of neurons to different degrees [126]. Furthermore, a new nanoconductor called MINDS (Macromolecular Infrared Nanotransducer for Deep brain Stimulation) was designed and reported to efficiently convert optical energy into thermal energy, thus making TRPV1 expression in deep brain neurons more sensitive to NIR laser irradiation. This can effectively avoid neuronal damage caused by stimulating TRPV1 proteins in deep brain regions with a strong NIR laser. It was reported that MINDS, located in the 5 mm deep region of the brain, produced much higher local temperatures than under NIR light passing through the scalp than the superficial brain region. Locally enhancing the response of neurons to NIR radiation may improve the efficiency of neural regulation within the safe power range of NIR radiation. As an extension of traditional photobiomodulation, SPN combined with NIR laser-activated neurons located in the deep brain led to the discovery of a new neural regulation pattern in animal experiments [127].

Recently, SPNs have been applied in the research of brain cognition-related diseases. Li et al. made a breakthrough in the treatment of depression-related cognitive disease with NIR photosensitive nanodrug delivery systems. Phenyl di-n-pentylphosphinate (PDPP) is a conjugated polymer that is a semiconductor polymer material. Using PDPP, DPPC, DSPE-PEG-NH_2_ and fasudil as raw materials, they prepared composite nanoparticles (NPs-F) by nanoprecipitation. Under NIR irradiation, the nanoparticles completed photothermal conversion in vivo, releasing fasudil and specifically activating Kv7.4 potassium channels. Electrophysiological studies showed that the firing frequency of dopaminergic neurons in the ventral tegmental area decreased significantly after treatment, suggesting that PDPP nanoparticles with excellent photothermal properties may be a future approach for the clinical treatment of depression (Fig. 5C) [128]. NIR laser pulses can instantaneously transport SPNs across the vascular barrier and accumulate in the designated sites due to the photoacoustic effects of the nanomaterial, as reported in the latest studies. They showed that after scanning the skulls for 10 min with an 840 nm laser pulse (50 mJ/cm^2^, 10 min), the accumulations of nanoparticles in the brains of mice increased significantly. This was the first time that nonfunctional NIR responsive nanomaterials could be accumulated through photoacoustic induction to target specific tissue sites with micron-scale precision, showing high efficiency and precision [129]. In the field of photoacoustic transformation, it was found that 1000–1350 nm NIR laser (21 mJ/cm^2^, ten 3-ns laser pulses at 1,030 nm over a 3-ms duration) can activate a new SPN with ultrasonic properties, which could target the primary motor cortex of C57BL/6 mice and change the local field potential and electromyography signals [130]. Although there are few reports of SPNs in the treatment of neurocognitive diseases, SPN combined with NIR laser may have a promising future as a phototherapy agent for treating brain disease owing to its high compatibility, flexibility and degradability.

#### Carbon-based nanomaterials

Carbon-based nanomaterials (CBNs) are attractive nanomaterials because of their structural diversity as well as unique photothermal properties. Various allotropes of carbon have been extensively studied for biomedical applications, such as carbon nanotubes (CNTs), graphene oxide (GO) and graphene quantum dots (GQDs) [131].

##### GO

In the field of neuroscience research, graphene is favoured for three applications. One is to use its electrical conductivity to promote the growth and differentiation of neurons. Akhavan's team prepared reduced graphene oxide nanomeshes (rGONMs) and applied them together with near-infrared lasers in experiments for human neural stem cell (hNSC) differentiation. During the experiments, hNSCs treated with an NIR laser and rGONMs differentiated more efficiently than those treated with graphene alone due to the stimulatory effects of the low-energy photoexcited electrons injected from the rGONM semiconductors into the cells [132]. Another application is to use the photothermal conversion effect of graphene materials to directly target neural lesions within the BBB. In a study of the treatment of AD, GO modified with thioflavin-S (ThS) was locally and remotely heated and decomposed amyloid protein aggregates under NIR laser irradiation, suggesting that modified GO may be a promising candidate for the photothermal treatment of AD [133].

**Fig. 6:**
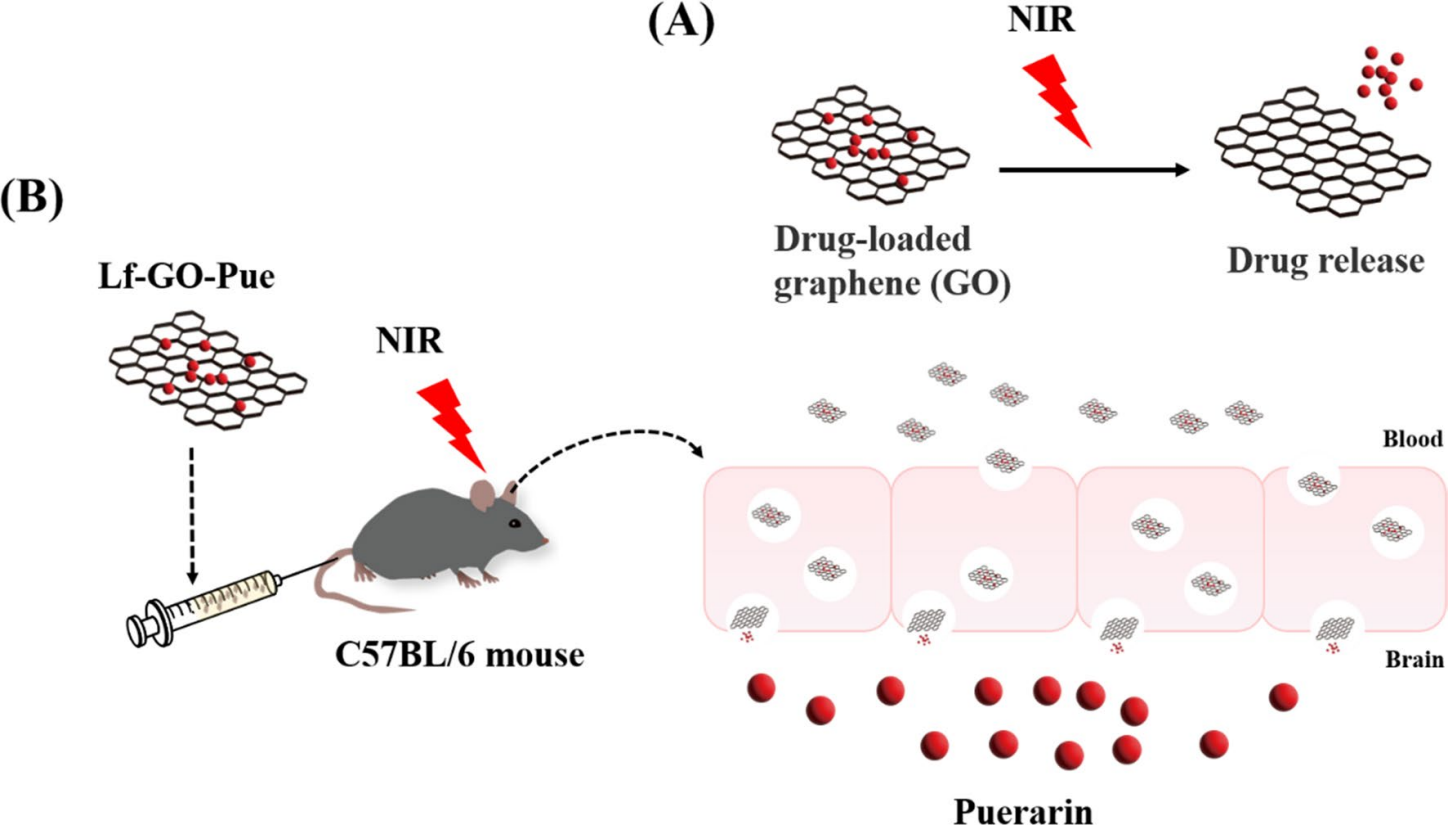
Treatment of cognitive dysfunction with carbon materials. **A** Scheme of drug release loaded on graphene sheet under NIR laser irradiation. **B** Schematic illustration of the nanocarrier (Lf-GO-Pue) for drug delivery to brain and across the blood brain barrier using NIR laser [139]. Copyright 2021, Royal Society of Chemistry and its reproduced permission granted

Moreover, graphene is an ideal candidate for drug delivery because of its enriched surface that is easily functionalized and coupled with different drugs, high chemical purity and free π electrons [134–136]. Otherwise, the oxidation and functionalization of graphene changes its photoelectric properties and increases its absorbance of visible and NIR light, which is conducive to photothermal drug delivery by NIR radiation [137, 138]. In addition, graphene can also be combined with other nanomaterials to make the nanoparticles more sensitive to the photothermal response of NIR light, resulting in more precise delivery of loaded drugs (Fig. 6A). A previous study demonstrated that PEG-functionalized GO can cause the loaded puerarin release from GO nanocarriers. Behavioural data showed that after the application of Lf-GO-Pue, the motor ability of the PD model mice (induced by MPTP drug injection) was improved, and the related symptoms were relieved. Puerarin-loaded lactoferrin functionalized graphene sheets (Lf-GO-Pue), which have been developed, have the potential to be a targeted drug delivery system for the combination of PD drug therapy and photothermal therapy (Fig. 6B) [139]. In addition to being used as drug transport carriers, graphene can also be used as a plasmid transfection carrier. Plasmid DNA can be loaded with neurotensin (NT)-conjugated polyethylenimine (PEI)-modified reduced GO nanoparticles. With the aid of NIR lasers (808 nm, 0.3 W/cm^2^, 30 min), membrane permeability was enhanced, and the intracellular degradation of nanoparticles was reduced, which enhanced the ability of the plasmid to target neurons and realized a high gene transfection rate of neurons [140]. In summary, these applications of graphene form the forefront of new research in the field of neurodegenerative diseases and neurotherapeutics [141].

##### CNT and GQD

The excellent conductivity of CNT/GQD combined with photothermal neural regulation may bring additional neural stimulation effects that are different from semiconductor polymer materials. NIR-responsive drug delivery and release of CNTs have been reported to be used in brain cancer therapy due to their biocompatibility and photothermal properties as well as their ability to penetrate the BBB [142, 143]. Furthermore, GQD refers to a single layer to several layers (3–10 layers) of zero-dimensional graphene sheets with transverse sizes less than 100 nm [144]. In addition to the related properties of graphene materials, GQDs also have the advantages of a single atomic layer, small lateral sizes, oxygen-rich surfaces and fluorescence characteristics [145], which make them suitable for loading therapeutic drugs or tracking drug release pathways [146–148]. However, the application of CNT/GQD-triggered photobiomodulation in neurocognitive diseases remains unknown, but due to their excellent biocompatibility and photothermal properties, CNT/GQD combined with NIR laser for photobiomodulation will have great potential applications in this field of research (Additional file 1).

## Limitations

Unfortunately, the penetration depth of NIR laser is restricted to the cerebral cortex or superficial brain areas, so it fails to modulate deep brain lesions, such as in the thalamus, which are closely associated with emotional disorders. Another disadvantage of NIR laser neuromodulation is the accidental overheating of brain tissue, which may cause inherent injury and inhibit neural activity, producing side effects in addition to normal regulation. Similarly, NIR laser, as a relatively safe physical therapy, is suitable for patients with cerebral ischemic diseases (such as ischemic stroke and neonatal hypoxic-ischemic brain injury) and less used for hemorrhagic stroke to avoid adverse consequences [149, 150]. Other studies have shown that high-power near-infrared light may cause retinal damage, and it is necessary to calculate the damage threshold temperatures and the maximum permissible exposure dose and time according to the individual situation [151, 152]. Besides, the application of nanoparticles also has some disadvantages. The biotoxicity of nanomedicine is related to the biological metabolism of nanoparticles leading to hepatotoxicity or nephrotoxicity, their applications are limited in animal experimental models only.

## Future prospective

Owing to effectiveness, direct and accurate on-site action, non-pharmacologically based therapies such as the strategies that we reviewed and presented herein are very innovative and promising for disease treatments. Indeed, photobiomodulation has been clinically used as an alternative method for treating brain diseases and can be classified as 1) transcranial photobiomodulation, which directly irradiates the head with red light or NIR laser and 2) an NIR-nanodelivery platform combined with photosensitive nanoparticles. Experimental data showed that these two strategies have good therapeutic effects in animals or even in human, and thus, photobiomodulation may become a novel breakthrough therapy with great potential for the treatment of cognitive impairment in conditions such as AD, PD and TBI. NIR drug delivery systems are commonly used in treating brain tumours but are very rarely applied for brain neuronal diseases. Although drug delivery systems using nanoparticles to treat cognitive disorders have been developed, most of them simply take advantage of the capacity of drug loading and slow release of nanoparticles. If the drugs can be loaded on nanoparticles containing NIR-responsive materials, the drugs can successfully escape the immune system and cross the BBB while still being released in targeted brain regions by artificial operation. At the same time, drug side effects can be avoided, and drug bioavailability can be improved. By maximizing drug bioavailability, the corresponding symptoms of cognitive impairment may be better improved, and the drug effects can be maintained for a longer period of time. However, as discussed above, the biocompatibility of nanoparticles is a big challenge and is an obstacle for its clinical use but fear assure is that biomaterials are being developed very fast and this gives a big hope towards clinical applications.

## Conclusion

Cognitive function is an advanced neurological function, which refers to the ability of our brains to form judgement and conclusions from information provided. As a common manifestation of brain diseases, cognitive dysfunction has a causal relationship with pathological changes in neurons and neural networks. To improve cognitive function, direct intervention in targeted encephalic regions or lesion sites may be the most effective therapeutic strategy. Compared with traditional systemic administrations of drugs, photobiomodulation is a novel and noninvasive physical therapy that utilizes the energy or photothermal effects of light. Photobiomodulation attracts much attention due to its benefits, ranging from safety, flexibility and operability. However, the combined therapy of photosensitive nanoparticles and lasers, which can accurately regulate neural activity and improve cognitive function, is a new direction in drug delivery development. Presently, it is far from clinical use, but undoubtedly, ongoing further research will make these strategies to be clinically available in the foreseeable future.

## Supplementary Information

**Additional file 1:** The supplementary material contains the Copyrights to the pictures in the body of the review, and the title may be called “Copyrights of relevant literature”.

## Data Availability

All other data are available from the corresponding authors upon reasonable request.

## Abbreviations

ATP: Adenosine triphosphate
ASD: Autism spectrum disorder
AD: Alzheimer's disease
Aβ: Amyloid-beta protein
Au: Gold material
AuNR: Gold nanorod
AuNP: Gold nanoparticles
AuNS: Gold nanostar
APH: Acylpeptide hydrolase
BBB: Blood brain barrier
CBF: Cerebral blood flow
CCO: Cytochrome C oxidase
Ca^2+^: Calcium ion
CBN: Carbon based nanomaterial
CNT: Carbon nanotubes
CW: Continuous wave
DCX: Docetaxel
DPPC: Dipalmitoyl phosphatidylcholine
DSPE: Distearoyl Phosphoethanolamine
EEG: Electroencephalogram
GABAergic: γ-Aminobutyrinergic
GO: Graphene oxide
GOD: Graphene quantum dots
GFAP: Glial fibrillary acidic portein
hNSC: Human neural stem cell
J/cm^2^: Joule per square centimeter
LDS: Lipid droplets
LED: Light emitting diode
LSPR: Localized surface plasmon resonance
Lf-GO-Pue: Puerarin loaded lactoferrin functionalized graphene sheets
MPTP: 1-Methyl-4-phenyl-1,2,3,6-tetrahy-dropyridine
mW: Milliwatt
mW/cm^2^: Milliwatts per square centimeter
MWM: Morris Water Maze
NIR: Near-infrared
NMDR: N-methyl-D-aspartate receptor
NT: Neurotensin
NH2: Amino end group
NPLT: Nano pulsed laser
PDA: Polydopamine
PDPP: Phenyl di-n-pentylphosphinate
PEG: Polyethylene glycol
PEI: Polyethylenimine
PW: Pulse wave
ROS: Reactive oxygen species
REV: Reverse evaporation
rGONMs: Reduced graphene oxide nanomeshes
SNc: Substantia nigra pars compacta
scFv: Single chain variable fragment
SPN: Semiconductor polymer nanoparticle
SVZ: Subependymal ventricular zone
TES: Transcranial electrical stimulation
TMS: Transcranial magnetic stimulation
TBI: Traumatic brain injury
TH +: Tyrosine hydroxylase positive
TRPV1: Transient Receptor Potential Vanilloid1
TFH: Thin film hydration
Tf: Transferrin
ThS: Thioflavin-S
