# Supplementary material for: Advances in photobiomodulation for cognitive improvement by near-infrared derived multiple strategies
Source: J Transl Med. 2023 Feb 22;21:135. doi: 10.1186/s12967-023-03988-w (PMC9945713; doi:10.1186/s12967-023-03988-w)
Supplement: Supplementary file 1 — Additional file 1: The supplementary material contains the Copyrights to the pictures in the body of the review, and the title may be called “Copyrights of relevant literature”. [file 12967_2023_3988_MOESM1_ESM.pdf]

Editing Certificate

This document certifies that the manuscript

Advances in Photobiomodulation for Cognitive Improvement by Near-Infrared Derived Multiple Strategies

prepared by the authors

Weitong Pan, Panmiao Liu, Daqing Ma, Jianjun Yang

was edited for proper English language, grammar, punctuation, spelling, and overall style by one or more of the highly qualified native English speaking editors at AJE.

This certificate was issued on **August 18, 2022** and may be verified on the AJE website using the verification code **524A-9986-550A-FF46-967A**.

Neither the research content nor the authors' intentions were altered in any way during the editing process. Documents receiving this certification should be English-ready for publication; however, the author has the ability to accept or reject our suggestions and changes. To verify the final AJE edited version, please visit our verification page at aje.com/certificate. If you have any questions or concerns about this edited document, please contact AJE at support@aje.com.

AJE provides a range of editing, translation, and manuscript services for researchers and publishers around the world.
For more information about our company, services, and partner discounts, please visit aje.com.

Creative Commons — Attribution

https://creativecommons.org/licenses/by/4.0/

Help us build a vibrant, collaborative global commons

Donate Now

This page is available in the following languages: English

Attribution 4.0 International (CC BY 4.0)

This is a human-readable summary of (and not a substitute for) the license. Disclaimer.

You are free to:

Free Cultural Works — APPROVED FOR

Share — copy and redistribute the material in any medium or format

Adapt — remix, transform, and build upon the material for any purpose, even commercially.

The licensor cannot revoke these freedoms as long as you follow the license terms.

Under the following terms:

Attribution — You must give appropriate credit, provide a link to the license, and indicate if changes were made. You may do so in any reasonable manner, but not in any way that suggests the licensor endorses you or your use.

No additional restrictions — You may not apply legal terms or technological measures that legally restrict others from doing anything the license permits.

Notices:

You do not have to comply with the license for elements of the material in the public domain or where your use is permitted by an applicable exception or limitation.

No warranties are given. The license may not give you all of the permissions necessary for your intended use. For example, other rights such as publicity, privacy, or moral rights may limit how you use the material.

Learn more about CC licensing, or use the license for your own material.

This content is freely available under simple legal terms because of Creative Commons, a non-profit that survives on donations. If you love this content, and love that it's free for everyone, please consider a donation to support our work.

Contribute today to Creative Commons

$5 | $15

$25 | $ Amount

Donate Now

CCC RightsLink®

? Help ∨ Live Chat

SPRINGER NATURE

Antibody-conjugated gold nanoparticles as nanotransducers for second near-infrared photo-stimulation of neurons in rats

Author: Jiansheng Liu et al
Publication: Nano Convergence
Publisher: Springer Nature
Date: Mar 21, 2022

Copyright © 2022, The Author(s)

Creative Commons

This is an open access article distributed under the terms of the Creative Commons CC BY license, which permits unrestricted use, distribution, and reproduction in any medium, provided the original work is properly cited.

You are not required to obtain permission to reuse this article.
To request permission for a type of use not listed, please contact Springer Nature

© 2022 Copyright - All Rights Reserved | Copyright Clearance Center, Inc. | Privacy statement | Terms and Conditions
Comments? We would like to hear from you. E-mail us at customercare@copyright.com

CCC | RightsLink®

Home | Help | Email Support | Sign in | Create Account

ACS Publications
Most Trusted. Most Cited. Most Read.

Gold Nanorods with Spatial Separation of CeO2 Deposition for Plasmonic-Enhanced Antioxidant Stress and Photothermal Therapy of Alzheimer's Disease

Author: Kezhen Ge, Yingfeng Mu, Miaoyan Liu, et al
Publication: Applied Materials
Publisher: American Chemical Society
Date: Jan 1, 2022

Copyright © 2022, American Chemical Society

PERMISSION/LICENSE IS GRANTED FOR YOUR ORDER AT NO CHARGE

This type of permission/license, instead of the standard Terms and Conditions, is sent to you because no fee is being charged for your order. Please note the following:

- Permission is granted for your request in both print and electronic formats, and translations.
- If figures and/or tables were requested, they may be adapted or used in part.
- Please print this page for your records and send a copy of it to your publisher/graduate school.
- Appropriate credit for the requested material should be given as follows: "Reprinted (adapted) with permission from {COMPLETE REFERENCE CITATION}. Copyright {YEAR} American Chemical Society." Insert appropriate information in place of the capitalized words.
- One-time permission is granted only for the use specified in your RightsLink request. No additional uses are granted (such as derivative works or other editions). For any uses, please submit a new request.

If credit is given to another source for the material you requested from RightsLink, permission must be obtained from that source.

BACK | CLOSE WINDOW

© 2022 Copyright - All Rights Reserved | Copyright Clearance Center, Inc. | Privacy statement | Terms and Conditions
Comments? We would like to hear from you. E-mail us at customercare@copyright.com

CCC Marketplace™

Order Number: 1221001
Order Date: 13 May 2022

Payment Information

Weitong Pan
pwt981013@163.com
Payment method: Invoice

Billing Address:
Miss Weitong Pan
The First Affiliated Hospita
l of Zhengzhou University
Zhengzhou
China

+86 13213191903
pwt981013@163.com

Customer Location:
Miss Weitong Pan
The First Affiliated Hospita
l of Zhengzhou University
Zhengzhou
China

Order Details

1. Biomaterials science

Billing Status: Open

Article: Targeted Graphene Oxide for Drug Delivery as a Therapeutic Nanoplatform against Parkinson’s Disease

**Order License ID**	1221001-1	**Type of use**	Republish in a journal/magazine
**Order detail status**	Completed	**Publisher**	Royal Society of Chemistry
**ISSN**	2047-4849	**Portion**	Image/photo/illustration

0.00 USD
Republication Permission

LICENSED CONTENT

**Publication Title**	Biomaterials science	**Publication Type**	e-Journal
**Article Title**	Targeted Graphene Oxide for Drug Delivery as a Therapeutic Nanoplatform against Parkinson’s Disease	**Start Page**	1705
		**End Page**	1715
		**Issue**	5
		**Volume**	9
**Author/Editor**	Kyōto Daigaku.Busshitsu-Saibō Tōgō Shisutemu Kyoten,Royal Society of Chemistry (Great Britain)	**URL**	http://pubs.rsc.org/en/journals/journalissues/bm
**Date**	01/01/2013		
**Language**	English		
**Country**	United Kingdom of Great Britain and Northern Ireland		
**Rightsholder**	Royal Society of Chemistry		

REQUEST DETAILS

**Portion Type**	Image/photo/illustration	**Distribution**	Worldwide
**Number of images / photos / illustrations**	1	**Translation**	Original language of publication
**Format (select all that apply)**	Print	**Copies for the disabled?**	No
**Who will republish the content?**	Publisher, STM	**Minor editing privileges?**	No
**Duration of Use**	Life of current edition	**Incidental promotional use?**	Yes
**Lifetime Unit Quantity**	Up to 499	**Currency**	USD
**Rights Requested**	Main product		

NEW WORK DETAILS

**Title**	Advances of Near-Infrared Photobiomodulation Integrating with Photosensitive Nanomaterials for Cognitive Improvement	**Publisher**	Wiley-VCH
		**Expected publication date**	2023-05-01
		**Expected size (number of pages)**	5
**Author**	Weitong Pan		
**Publication**	Advanced science		

ADDITIONAL DETAILS

**The requesting person / organization to appear on the license**	Weitong Pan

REUSE CONTENT DETAILS

**Title, description or numeric reference of the portion(s)**	Figure 1	**Title of the article/chapter the portion is from**	Targeted Graphene Oxide for Drug Delivery as a Therapeutic Nanoplatform against Parkinson’s Disease
**Editor of portion(s)**	Xiong, Sha; Luo, Jingshan; Wang, Qun; Li, Zhongjun; Li, Juntong; Liu, Qiao; Gao, Liqian; Fang, Shu-Huan; Li, Yunyong; Pan, Huafeng; Wang, Hong; Zhang, Yongbin; Wang, Qi; Chen, Xiao-jia; Chen, Tongkai	**Author of portion(s)**	Xiong, Sha; Luo, Jingshan; Wang, Qun; Li, Zhongjun; Li, Juntong; Liu, Qiao; Gao, Liqian; Fang, Shu-Huan; Li, Yunyong; Pan, Huafeng; Wang, Hong; Zhang, Yongbin; Wang, Qi; Chen, Xiao-jia; Chen, Tongkai
**Volume of serial or monograph**	9		
**Page or page range of portion**	1705-1715	**Issue, if republishing an article from a serial**	5
		**Publication date of portion**	2021-03-10

Total Items: 1

Subtotal: 0.00 USD

Order Total: 0.00 USD

JOHN WILEY AND SONS LICENSE
TERMS AND CONDITIONS

Jan 18, 2022

This Agreement between Weitong Pan ("You") and John Wiley and Sons ("John Wiley and Sons") consists of your license details and the terms and conditions provided by John Wiley and Sons and Copyright Clearance Center.

License Number	5232211476269
License date	Jan 18, 2022
Licensed Content Publisher	John Wiley and Sons
Licensed Content Publication	Advanced Functional Materials

Licensed Content Title	Photothermal Modulation of Depression-Related Ion Channel Function through Conjugated Polymer Nanoparticles
Licensed Content Author	Boying Li, Yiying Wang, Dong Gao, et al
Licensed Content Date	Mar 3, 2021
Licensed Content Volume	31
Licensed Content Issue	19
Licensed Content Pages	9
Type of use	Journal/Magazine
Requestor type	Publisher (STM Signatory)
STM publisher name	Elsevier

Is the reuse sponsored by or associated with a pharmaceutical or medical products company?	no
Format	Print and electronic
Portion	Figure/table
Number of figures/tables	1
Will you be translating?	No
Circulation	1 - 29
Title of new article	A review of the pathogenesis of cognitive dysfuction and the development of corresponding photobiomodulation methods
Lead author	Weitong Pan

Title of targeted journal	Engineered Regeneration
Publisher	Elsevier
Expected publication date	Oct 2023
Order reference number	1
Portions	Figure 1
Requestor Location	Weitong Pan<br>The First Affiliated Hospital of Zhengzhou University<br><br>Zhengzhou, other<br>China<br>Attn: Miss Weitong Pan
Publisher Tax ID	EU826007151
Total	0.00 USD

TERMS AND CONDITIONS

This copyrighted material is owned by or exclusively licensed to John Wiley & Sons, Inc. or one of its group companies (each a"Wiley Company") or handled on behalf of a society with which a Wiley Company has exclusive publishing rights in relation to a particular work (collectively "WILEY"). By clicking "accept" in connection with completing this licensing transaction, you agree that the following terms and conditions apply to this transaction (along with the billing and payment terms and conditions established by the Copyright Clearance Center Inc., ("CCC's Billing and Payment terms and conditions"), at the time that you opened your RightsLink account (these are available at any time at http://myaccount.copyright.com).

Terms and Conditions

- The materials you have requested permission to reproduce or reuse (the "Wiley Materials") are protected by copyright.

- You are hereby granted a personal, non-exclusive, non-sub licensable (on a stand-alone basis), non-transferable, worldwide, limited license to reproduce the Wiley Materials for the purpose specified in the licensing process. This license, **and any CONTENT (PDF or image file) purchased as part of your order,** is for a one-time use only and limited to any maximum distribution number specified in the license. The first instance of republication or reuse granted by this license must be completed within two years of the date of the grant of this license (although copies prepared before the end date may be distributed thereafter). The Wiley Materials shall not be used in any other manner or for any other purpose, beyond what is granted in the license. Permission is granted subject to an appropriate acknowledgement given to the author, title of the material/book/journal and the publisher. You shall also duplicate the copyright notice that appears in the Wiley publication in your use of the Wiley Material. Permission is also granted on the understanding that nowhere in the text is a previously published source acknowledged for all or part of this Wiley Material. Any third party content is expressly excluded from this permission.

- With respect to the Wiley Materials, all rights are reserved. Except as expressly granted by the terms of the license, no part of the Wiley Materials may be copied,

modified, adapted (except for minor reformatting required by the new Publication), translated, reproduced, transferred or distributed, in any form or by any means, and no derivative works may be made based on the Wiley Materials without the prior permission of the respective copyright owner.**For STM Signatory Publishers clearing permission under the terms of the STM Permissions Guidelines only, the terms of the license are extended to include subsequent editions and for editions in other languages, provided such editions are for the work as a whole in situ and does not involve the separate exploitation of the permitted figures or extracts,** You may not alter, remove or suppress in any manner any copyright, trademark or other notices displayed by the Wiley Materials. You may not license, rent, sell, loan, lease, pledge, offer as security, transfer or assign the Wiley Materials on a stand-alone basis, or any of the rights granted to you hereunder to any other person.

- The Wiley Materials and all of the intellectual property rights therein shall at all times remain the exclusive property of John Wiley & Sons Inc, the Wiley Companies, or their respective licensors, and your interest therein is only that of having possession of and the right to reproduce the Wiley Materials pursuant to Section 2 herein during the continuance of this Agreement. You

agree that you own no right, title or interest in or to the Wiley Materials or any of the intellectual property rights therein. You shall have no rights hereunder other than the license as provided for above in Section 2. No right, license or interest to any trademark, trade name, service mark or other branding ("Marks") of WILEY or its licensors is granted hereunder, and you agree that you shall not assert any such right, license or interest with respect thereto

- NEITHER WILEY NOR ITS LICENSORS MAKES ANY WARRANTY OR REPRESENTATION OF ANY KIND TO YOU OR ANY THIRD PARTY, EXPRESS, IMPLIED OR STATUTORY, WITH RESPECT TO THE MATERIALS OR THE ACCURACY OF ANY INFORMATION CONTAINED IN THE MATERIALS, INCLUDING, WITHOUT LIMITATION, ANY IMPLIED WARRANTY OF MERCHANTABILITY, ACCURACY, SATISFACTORY QUALITY, FITNESS FOR A PARTICULAR PURPOSE, USABILITY, INTEGRATION OR NON-INFRINGEMENT AND ALL SUCH WARRANTIES ARE HEREBY EXCLUDED BY WILEY AND ITS LICENSORS AND WAIVED BY YOU.

- WILEY shall have the right to terminate this Agreement immediately upon breach of this Agreement by you.

- You shall indemnify, defend and hold harmless WILEY, its Licensors and their respective directors, officers, agents and employees, from and against any actual or threatened claims, demands, causes of action or proceedings arising from any breach of this Agreement by you.

- IN NO EVENT SHALL WILEY OR ITS LICENSORS BE LIABLE TO YOU OR ANY OTHER PARTY OR ANY OTHER PERSON OR ENTITY FOR ANY SPECIAL, CONSEQUENTIAL, INCIDENTAL, INDIRECT, EXEMPLARY OR PUNITIVE DAMAGES, HOWEVER CAUSED, ARISING OUT OF OR IN CONNECTION WITH THE DOWNLOADING, PROVISIONING, VIEWING OR USE OF THE MATERIALS REGARDLESS OF THE FORM OF ACTION, WHETHER FOR BREACH OF CONTRACT, BREACH OF WARRANTY, TORT, NEGLIGENCE, INFRINGEMENT OR OTHERWISE (INCLUDING, WITHOUT LIMITATION, DAMAGES BASED ON LOSS OF PROFITS, DATA, FILES, USE, BUSINESS OPPORTUNITY OR CLAIMS OF THIRD PARTIES), AND WHETHER OR NOT THE PARTY HAS BEEN ADVISED OF THE POSSIBILITY OF SUCH DAMAGES. THIS LIMITATION SHALL APPLY NOTWITHSTANDING ANY FAILURE OF ESSENTIAL PURPOSE OF ANY LIMITED REMEDY PROVIDED HEREIN.

- Should any provision of this Agreement be held by a court of competent jurisdiction to be illegal, invalid, or unenforceable, that provision shall be deemed amended to achieve as nearly as possible the same economic effect as the original provision, and the legality, validity and enforceability of the remaining provisions of this Agreement shall not be affected or impaired thereby.

- The failure of either party to enforce any term or condition of this Agreement shall not constitute a waiver of either party's right to enforce each and every term and condition of this Agreement. No breach under this agreement shall be deemed waived or excused by either party unless such waiver or consent is in writing signed by the party granting such waiver or consent. The waiver by or consent of a party to a breach of any provision of this Agreement shall not operate or be construed as a waiver of or consent to any other or subsequent breach by such other party.

- This Agreement may not be assigned (including by operation of law or otherwise) by you without WILEY's prior written consent.

- Any fee required for this permission shall be non-refundable after thirty (30) days from receipt by the CCC.

- These terms and conditions together with CCC's Billing and Payment terms and conditions (which are incorporated herein) form the entire agreement between you and WILEY concerning this licensing transaction and (in the absence of fraud) supersedes all prior agreements and representations of the parties, oral or written. This Agreement may not be amended except in writing signed by both parties. This Agreement shall be binding upon and inure to the benefit of the parties' successors, legal representatives, and authorized assigns.

- In the event of any conflict between your obligations established by these terms and conditions and those established by CCC's Billing and Payment terms and conditions, these terms and conditions shall prevail.

- WILEY expressly reserves all rights not specifically granted in the combination of (i) the license details provided by you and accepted in the course of this licensing transaction, (ii) these terms and conditions and (iii) CCC's Billing and Payment terms and conditions.

- This Agreement will be void if the Type of Use, Format, Circulation, or Requestor Type was misrepresented during the licensing process.

- This Agreement shall be governed by and construed in accordance with the laws of the State of New York, USA, without regards to such state's conflict of law rules. Any legal action, suit or proceeding arising out of or relating to these Terms and Conditions or the breach thereof shall be instituted in a court of competent jurisdiction in New York County in the State of New York in the United States of America and each party hereby consents and submits to the personal jurisdiction of such court, waives any objection to venue in such court and consents to service of process by registered or certified mail, return receipt requested, at the last known address of such party.

WILEY OPEN ACCESS TERMS AND CONDITIONS

Wiley Publishes Open Access Articles in fully Open Access Journals and in Subscription journals offering Online Open. Although most of the fully Open Access journals publish open access articles under the terms of the Creative Commons Attribution (CC BY) License only, the subscription journals and a few of the Open Access Journals offer a choice of Creative Commons Licenses. The license type is clearly identified on the article.

The Creative Commons Attribution License

The Creative Commons Attribution License (CC-BY) allows users to copy, distribute and transmit an article, adapt the article and make commercial use of the article. The CC-BY license permits commercial and non-

Creative Commons Attribution Non-Commercial License

The Creative Commons Attribution Non-Commercial (CC-BY-NC)License permits use, distribution and reproduction in any medium, provided the original work is properly cited and is not used for commercial purposes.(see below)

Creative Commons Attribution-Non-Commercial-NoDerivs License

The Creative Commons Attribution Non-Commercial-NoDerivs License (CC-BY-NC-ND) permits use, distribution and reproduction in any medium, provided the original work is properly cited, is not used for commercial purposes and no modifications or adaptations are made. (see below)

Use by commercial "for-profit" organizations

Use of Wiley Open Access articles for commercial, promotional, or marketing purposes requires further explicit permission from Wiley and will be subject to a fee.

Further details can be found on Wiley Online Library
http://olabout.wiley.com/WileyCDA/Section/id-410895.html

Other Terms and Conditions:

v1.10 Last updated September 2015

Questions? customercare@copyright.com or +1-855-239-3415 (toll free in the US) or +1-978-646-2777.
